# Rift Valley Fever Virus Infection in Golden Syrian Hamsters

**DOI:** 10.1371/journal.pone.0116722

**Published:** 2015-01-21

**Authors:** Dionna Scharton, Arnaud J. Van Wettere, Kevin W. Bailey, Zachary Vest, Jonna B. Westover, Venkatraman Siddharthan, Brian B. Gowen

**Affiliations:** 1 Department of Animal, Dairy, and Veterinary Sciences, Utah State University, Logan, Utah, United States of America; 2 Institute for Antiviral Research, Utah State University, Logan, Utah, United States of America; 3 School of Veterinary Medicine, Utah State University, Logan, Utah, United States of America; 4 Utah Veterinary Diagnostic Laboratory, Logan, Utah, United States of America; George Mason University, UNITED STATES

## Abstract

Rift Valley fever virus (RVFV) is a formidable pathogen that causes severe disease and abortion in a variety of livestock species and a range of disease in humans that includes hemorrhagic fever, fulminant hepatitis, encephalitis and blindness. The natural transmission cycle involves mosquito vectors, but exposure can also occur through contact with infected fluids and tissues. The lack of approved antiviral therapies and vaccines for human use underlies the importance of small animal models for proof-of-concept efficacy studies. Several mouse and rat models of RVFV infection have been well characterized and provide useful systems for the study of certain aspects of pathogenesis, as well as antiviral drug and vaccine development. However, certain host-directed therapeutics may not act on mouse or rat pathways. Here, we describe the natural history of disease in golden Syrian hamsters challenged subcutaneously with the pathogenic ZH501 strain of RVFV. Peracute disease resulted in rapid lethality within 2 to 3 days of RVFV challenge. High titer viremia and substantial viral loads were observed in most tissues examined; however, histopathology and immunostaining for RVFV antigen were largely restricted to the liver. Acute hepatocellular necrosis associated with a strong presence of viral antigen in the hepatocytes indicates that fulminant hepatitis is the likely cause of mortality. Further studies to assess the susceptibility and disease progression following respiratory route exposure are warranted. The use of the hamsters to model RVFV infection is suitable for early stage antiviral drug and vaccine development studies.

## Introduction

Rift Valley fever (RVF) is a zoonotic, arthropod-borne illness that typically manifests as an acute febrile and hepatic disease in ungulates and humans. RVF is of notable public health importance due to its severity, recurrent outbreaks and progressive geographic distribution [1–3]. The etiological agent, Rift Valley fever virus (RVFV), is a member of the *Bunyaviridae* family and the genus *Phlebovirus*. The virus has a tripartite single-stranded RNA genome which encodes 5 proteins using an ambisense coding strategy [2, 4]. It can be transmitted by a variety of mosquito species, but is also spread via contact with infected fluids and tissues [5].

Susceptibility of livestock to RVFV infection varies greatly depending on the viral strain, and the species and age of the infected animal [6]. Hepatic necrosis with consequent increase in liver enzyme, and high viremia are characteristic of severe acute lethal infection in ruminants. In humans, the virus has an incubation period of 2–6 days, after which flu-like clinical signs appear and typically last 2–7 days after onset of illness [4, 5, 7]. Symptoms are generally described as an abrupt onset of fever, chills, and lethargy with 1–3% of cases progressing to more serious forms of disease including hemorrhagic syndrome, acute-onset hepatitis, delayed-onset encephalitis with long-term neurologic deficits, and retinal vasculitis and macular lesions which can result in varying degrees of blindness [8]. In severe cases of RVF, the fatality rate is 10–20%, but in recent outbreaks it has climbed as high as 40% [9]. Currently, no FDA approved vaccines or antiviral therapies for the prevention or treatment of RVF exist. Consequently, the development of animal models to better understand the disease is of increasing importance when considering the threat RVFV presents to public and animal health and the potential for importation into the US or other naïve regions of the world that harbor competent mosquito vector populations [10–12].

The key pathological features of RVFV infection vary widely among animal species and humans. Typically, RVFV infection that results in severe disease is characterized by hepatocellular necrosis [5, 13]. Because of the greater biohazard risk and “Select Agent” status of RVFV, a more accessible hamster model for RVF is based on challenge with the related Punta Toro virus (PTV), a BSL-2 agent, has been used for pathogenesis and antiviral studies [14]. Although the hamster PTV infection model has proved useful for reproducing certain features of severe human and animal RVFV infections where hepatic disease is a prominent pathological feature, the animals fail to develop encephalitis [15, 16]. Recently, a detailed characterization describing the pathogenesis of RVFV infection in BALB/c mice reported hepatitis and encephalitis consistent with severe human RVFV infection [17]. Additionally, a study using three different inbred strains of rats infected with RVFV by both aerosol and sub-cutaneous (s.c.) routes demonstrated remarkable differences in disease progression and lethality [18]. Wistar-Furth rats were the only strain to develop and succumb to acute hepatic disease following aerosol exposure. ACI and Lewis rat strains both developed fatal encephalitis after aerosol challenge, but with varying degrees of susceptibility to RVFV; remarkably, Lewis rats are refractory to s.c. challenge [18]. These differences are consistent with the varying clinical disease presentations observed in humans. Although these murine and rat RVFV models are useful systems to evaluate most vaccine and antiviral drug candidates, certain therapeutic platforms, particularly those directed at host targets, may have little to no activity in mice or rats. For example, consensus IFN, an FDA-licensed recombinant protein therapeutic, was evaluated in the hamster PTV model because it does not cross-react with the mouse system [19, 20].

Hamsters models are becoming more widely used in infectious disease research, with the biggest increase observed in studies of viral infections [21]. Several New World arenaviruses that are cleared readily by mice shortly after challenge cause acute hemorrhagic fever-like disease in hamsters [22, 23]. Other examples of hamster models where comparable disease is absent in mice are based on challenge with Andes hantavirus and yellow fever virus [23–25], and these and other hamster models have been useful tools for antiviral efficacy and pathogenesis studies [26]. In the case of West Nile virus infection, disease in hamsters was more representative of the human condition in terms of neurologic disease compared to mice [27]. Thus, the upticks in studies employing hamster viral infection models is likely due to a combination of the aforementioned examples, and the relatively low cost and ease of handling the animals.

We recently evaluated a promising broad-spectrum antiviral drug candidate and adenovirus vectored human consensus IFN in a model of RVFV infection in hamsters [13, 28]. Limited details describing RVFV infection and disease in hamsters have previously been reported [29–34]. Here, we present linked virologic, liver enzyme, and pathologic findings during the course of RVFV infection in golden Syrian hamsters challenged s.c. with the pathogenic ZH501 strain of RVFV to gain insights into the natural history of disease in this small animal model of RVF.

## Materials and Methods

### Virus and cells

The molecular clone of RVFV, strain ZH501, was obtained from Dr. Stuart Nichol (CDC, Atlanta, GA). The virus stock (1.1 × 10^8^ plaque-forming units (PFU)/ml); 1 passage in BSRT7 cells, 3 passages in Vero E6 cells) used was from a clarified cell culture lysate preparation and was inoculated by subcutaneous (s.c.) injection (ventral, right side of abdomen). The African green monkey kidney cell line, Vero 76, was purchased from the American Type Culture Collection (ATCC) (Manassas, VA) and maintained in minimal essential medium (MEM) supplemented with 10% heat-inactivated fetal bovine serum (FBS) (Thermo Fisher Scientific HyClone, Logan, UT).

### Plaque assay

To determine the PFU/ml of our virus stock, Vero 76 cells were seeded in 6-well plates and grown to ~90% confluency. The virus was serially diluted in ten-fold dilutions and samples were added in duplicate to the plates. The plates were then incubated for 1 h at 37°C with 5% CO_2_, with rocking every 10 minutes to ensure full exposure to the monolayer. After incubation, the viral inoculum was removed from the wells, washed with 2 ml DPBS, and subsequently covered with 2 ml of a primary overlay medium consisting of 2% sea plaque agarose (SeaKem, ME), 2X MEM containing 4% FBS and 0.5% gentamicin. The plates were then incubated at 37°C with 5% CO_2_ for 4 days. Plaques were resolved by the addition of 1 ml of sterile Neutral Red (NR) to the primary overlay. Following incubated at 37°C for 2 h, the NR was removed and plaques counted after 2–3 h when they became readily visible. A second count was performed the next day to confirm plaques that were difficult to discern on day 4.

### Animals and ethics regulation

Female 90–115 g golden Syrian hamsters (The Charles River Laboratory, Willimantic, CT) were quarantined for 7 days prior to challenge and fed standard Harlan lab block and tap water *ad libitum*. All animal procedures complied with USDA guidelines and were conducted at the AAALAC-accredited Laboratory Animal Research Center at Utah State University under protocol # 2011, approved by the Utah State University Institutional Animal Care and Use Committee.

### Titration of RVFV in hamsters

To determine the most appropriate RVFV challenge dose for the natural history study, hamsters (n = 5–6/group) were challenged by s.c. injection with varying log_10_ dilutions of RVFV spanning 6 orders of magnitude. The s.c. challenge was intended to simulate natural mosquito-borne transmission. The animals were observed twice/day over a period of 15 days for morbidity and mortality, and weighed every 3 days starting on the day of challenge. Pain and distress was to be minimized as much as possible by using an alternative endpoint for euthanasia defined as unresponsiveness. Although animals may lose or fail to gain weight, appear lethargic, present with ruffled fur and hunched posture during the infection, these signs and symptoms are not hard-fast indicators of disease outcome as some animals can recover from illness. Due to the peracute nature of the disease, the criterion by CO_2_ asphyxiation was not met by any of the animals in this study. As pain alleviation would interfere with end point measurements, pain-relieving compounds were not used.

### Natural history of RVFV infection in hamsters

Based on the titration study, challenge doses of 10 PFU or 1 PFU were selected to evaluate the progression and tissue tropism of RVFV infection. Hamsters (n = 4–6/group) were selected for sacrifice on days 1 through 4 post s.c. challenge with RVFV. Tissue samples were collected (pancreas, spleen, liver, lung, brain, large intestine, kidney, adrenal gland, and eye) for virus titer determination, histopathology and immunohistochemistry (IHC) analysis, as described below. Whole blood was collected for clinical chemistry analysis, and serum was assayed for viral load and kinetic alanine aminotransferase (ALT) levels.

### Cell culture infectious dose assay

For the natural history of disease study, viral titers in serum and tissues were measured using an infectious 50% cell culture infectious dose (CCID_50_) assay. Tissue samples were homogenized in a fixed volume of MEM and the homogenates and serum was serially diluted and added to quadruplicate wells of Vero cell monolayers in 96-well microplates. The viral cytopathic effect (CPE) was determined 3–4 days post-plating, and the 50% endpoints were calculated as described [35]. The lower limit of detection for serum samples was 1.75 log_10_ CCID_50_/ml and the lower limit of detection for tissues was generally in the range of 2–3 log_10_ CCID_50_/g.

### Kinetic serum alanine aminotransferase (ALT) determinations

Detection of ALT in serum is an indirect method for evaluating hepatocellular injury. Serum ALT concentrations were measured using the ALT (SGPT) Reagent Set purchased from Pointe Scientific, Inc. (Lincoln Park, MI) per the manufacturer’s recommendations. The reagent volumes were adjusted for analysis on 96-well microplates.

### Histopathology

Tissue samples of the pancreas, spleen, liver, lung, kidney, adrenal gland, large intestine, brain and eye were obtained at prescribed necropsy times and preserved for 3 weeks in 10% neutral buffered formalin. The samples were subsequently sent to the Utah Veterinary Diagnostic Laboratory (Logan, UT) for blinded histopathology examination and analysis by a board certified veterinary pathologist.

### Immunohistochemical staining

Based on viral burden in the tissues and histopathology review, replicate tissue sections from a representative animal per sacrifice group were selected for immunohistochemical (IHC) staining. The sections were deparaffinized and rehydrated by standard histological procedures with xylene-ethanol, descending grades of alcohol, and distilled water. Briefly, sections were immersed in DakoCytomation Target Retrieval Solution (Dako Corp., Carpinteria, CA), boiled at 125°C for 4 minutes in a decloaking chamber (Biocare Medical, Concord, CA), permeabilized with 0.5% X-100 in PBS, and exposed to a peroxide block using 3% hydrogen peroxide. Slides were then incubated in 10% normal goat serum (NGS) and 0.2% Triton X-100 in PBS for 1 hour, and subsequently incubated with a mouse anti-RVFV Ab (1:1000; RVF MP-12 mouse hyperimmune ascites fluid provided by Dr. Robert Tesh, World Reference Center for Emerging Viruses and Arboviruses, University of Texas Medical Branch, Galveston, TX) for 24 hours at room temperature. Secondary antibody using goat anti-mouse HRP (1:200; Sigma-Aldrich, St. Louis, MO) was applied to the slides for 1 hour, then incubated for 15 minutes using Immpact NovaRed substrate (Vector Laboratories, Burlingame, CA), and counterstained with hematoxylin QS nuclear counterstain (Vector Laboratories). Lastly, sections were dehydrated in ascending grades of alcohol, passed in xylene and permanently mounted with non-aqueous mounting medium VectaMount (Vector Laboratories). The stained slides were sent to the Utah Veterinary Diagnostic Laboratory for IHC/histopathology examination and analysis by a board certified veterinary pathologist.

## Results

### Susceptibility of hamsters to RVFV

**Figure 1:**
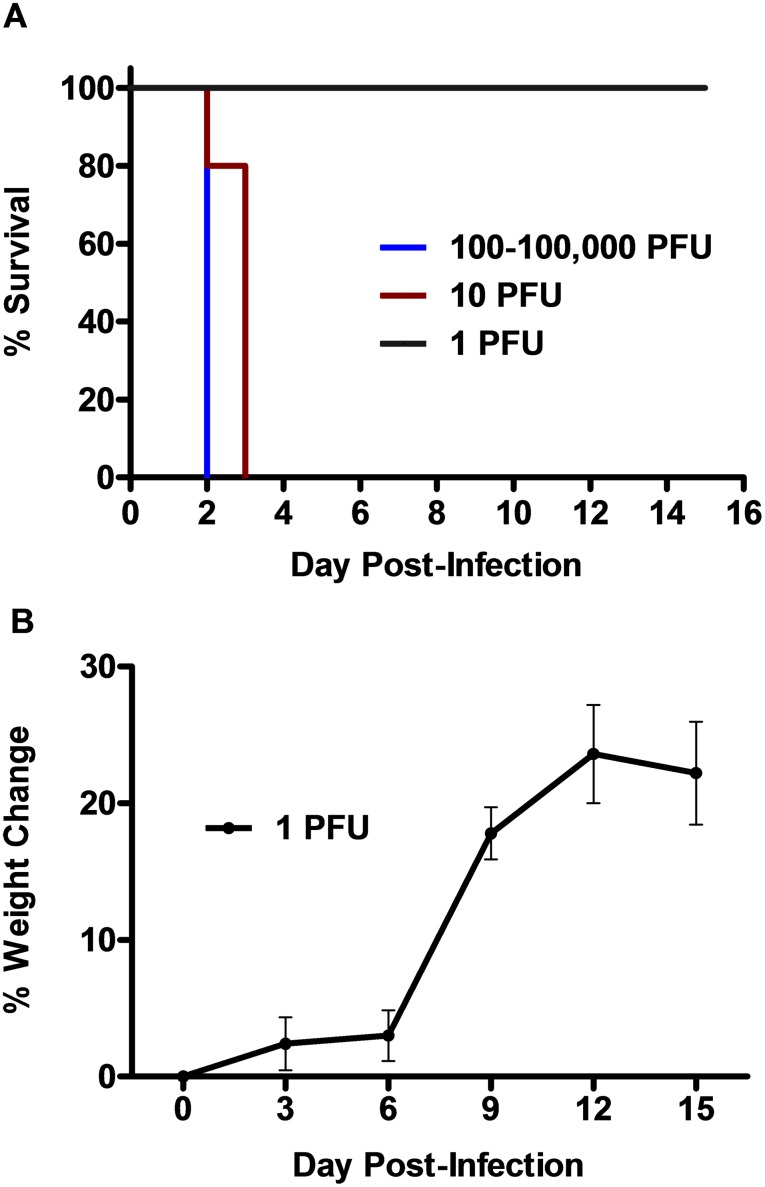
RVFV challenge of golden Syrian hamsters is rapidly lethal. Groups of 5–6 hamsters were infected s.c. with 0.2 ml of viral inoculum containing the indicated PFU of RVFV. Mortality was monitored over a 15-day period. A) Percent survival and B) mean % change in weight of surviving animals relative to respective day 0 weights measured every 3^rd^ day are shown.

The initial titration of the ZH501 strain of RVFV in golden Syrian hamsters revealed a rapid disease progression which was predominately lethal. Clinical signs of illness including lethargy, ruffled fur, and hunched posture were observed in many of the animals by day 2 post-infection (p.i.). The virus was uniformly lethal within 2–3 days following s.c. route inoculation at doses of 10 PFU or greater (Fig. 1A). Only the animals that received the lowest infectious dose of approximately 1 PFU (based on plaque titration in Vero 76 cells) survived the challenge. Although none of these animals succumbed to infection, the marked increase in weight beginning day 6 p.i. suggests that the animals were likely exhibiting some degree of illness early during the course of infection (Fig. 1B).

### Characterization of RVFV disease progression

Based on the titration experiment demonstrating high susceptibility of hamsters to s.c. RVFV infection, we next challenged animals with either 10 or 1 PFU of RVFV to assess the natural history of disease. Subsets of animals were sacrificed on day’s 1—4 p.i., to examine the development of viremia, tissue titers, ALT, and histopathology in a temporal fashion. Because all animals receiving 10 PFU in the titration study succumbed by day 3 p.i., this portion of the study was designed to have only a day 1 and 2 sacrifice. For the animals challenged with 1 PFU, several animals scheduled for sacrifice on day 3 (3 of 6 hamsters) and 4 (2 of 6 hamsters) succumbed prior to the time of sacrifice. The threshold for lethality appears to be very close to 1 PFU, and thus the lack of mortality with the 1 PFU challenge in the titration study is likely due to experimental variability in the preparation of the challenge stock.

**Figure 2:**
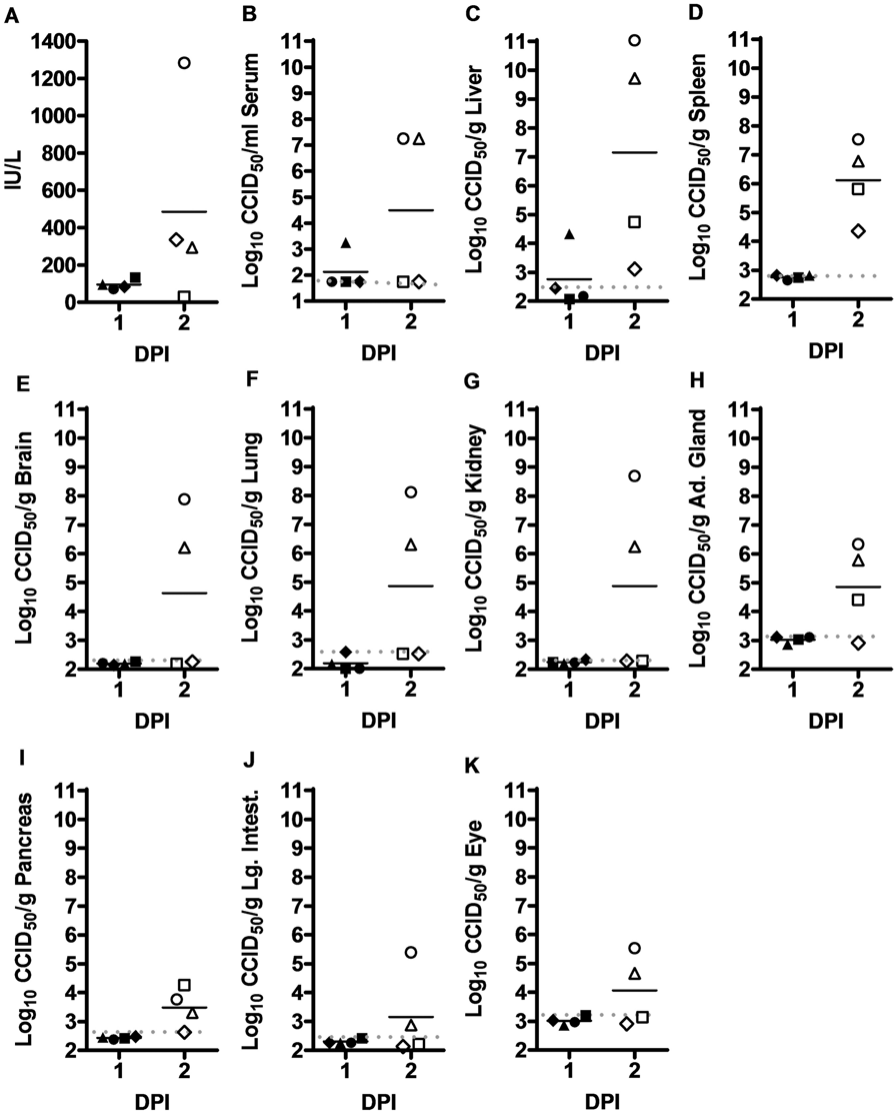
Temporal analysis of ALT levels and virus titers in hamsters challenged s.c. with 10 PFU of RVFV. Groups of 4 animals were sacrificed on the specified days post-infection for analysis of A) serum ALT concentration, and B) serum, C) liver, D) spleen, E) brain, F) lung, G) kidney, H) adrenal gland, I) pancreas, J) large intestine, and K) eye virus titers. Unique symbols represent values for the same animal across all parameters assessed and the gray dotted lines represents the limits of detection for each tissue or serum. DPI, day post-infection.

In the 10 PFU challenge group, serum ALT was not elevated until day 2 p.i. in one hamster and slightly elevated in two others (Fig. 2A). In one animal, low level viremia and elevated viral loads in the liver were detected as early as day 1 p.i. (Fig. 2B, C). In the day 2 p.i. cohort of animals two hamsters had virus in all tissues examined, with low-level or undetectable virus burden in the other two animals (Fig. 2B–K).

**Figure 3:**
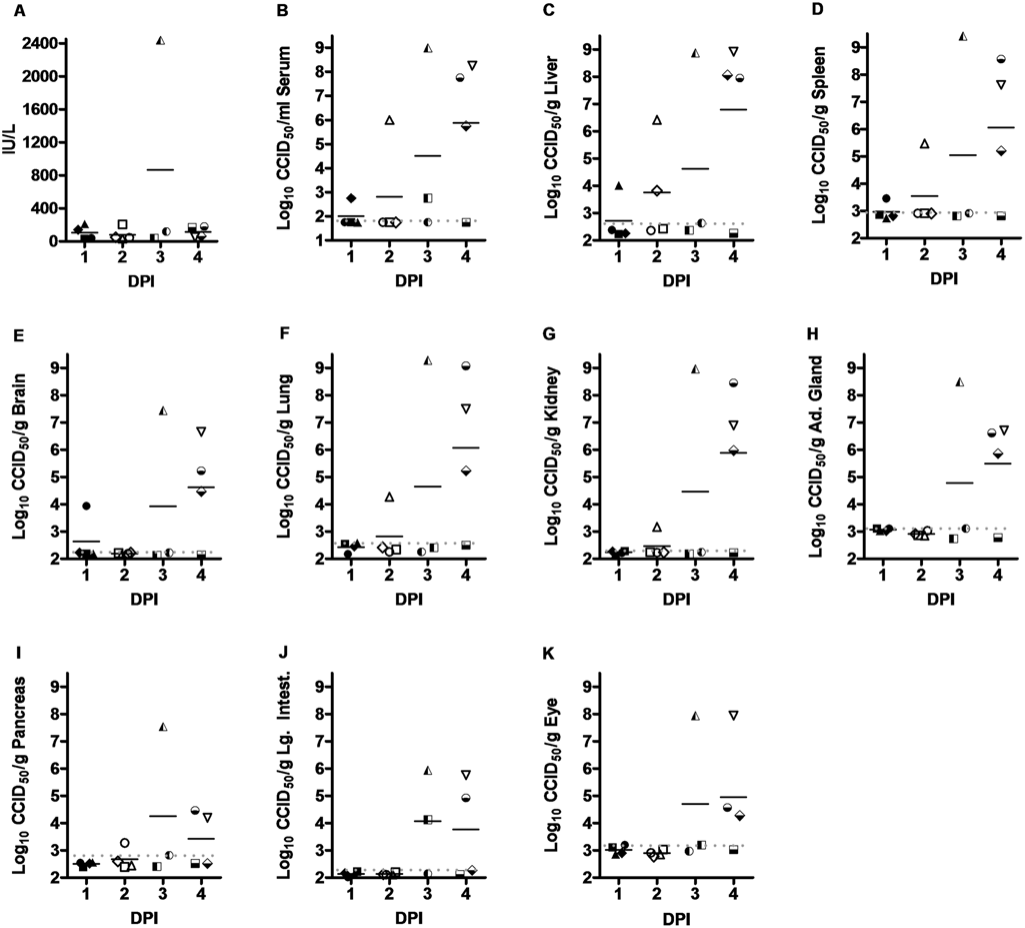
Temporal analysis of ALT levels and virus titers in hamsters challenged with 1 PFU of RVFV. Groups of 3–4 animals were sacrificed on the specified days post-infection for analysis of A) serum ALT concentration, and B) serum, C) liver, D) spleen, E) brain, F) lung, G) kidney, H) adrenal gland, I) pancreas, J) large intestine, and K) eye virus titers. Several hamsters succumbed prior to their designated time of sacrifice (3 in the day 3 sacrifice group and 2 in the day 4 group) and thus were not included in the analysis. The limits of detection are indicated by the grey dotted lines. Unique symbols represent values for the same animal across all parameters assessed. DPI, day post-infection.

When the RVFV challenge dose was reduced to 1 PFU, ALT concentration was dramatically elevated on day 3 (>2300 IU/L) in the only surviving hamster which also had significant liver virus titers (Fig. 3A, C). Despite substantial viral loads in the livers of 3 of the 4 animals in the day 4 group, ALT levels were not significantly elevated. Little to no virus was detected in the serum 24 h after challenge and only one of four hamsters had viremia in the 48 h cohort (Fig. 3B). By day 3 p.i., 2 of 3 surviving animals had measurable virus, and on day 4 p.i., 3 of 4 hamsters had remarkable viremia. In general, the day 3 and day 4 animals with high viremias had substantial viral loads in all tissues examined; the highest levels of virus were found in the liver, spleen, lung, kidney and adrenal gland (between 8.5–9.4 log_10_ CCID_50_/g), and significant amounts were detected in the brain, pancreas, large intestine and the eye (between 5.9–7.9 log_10_ CCID_50_/g) (Fig. 3C–K). Due to the expiration of several hamsters prior to their designated time of sacrifice, it is important to note that data from the animals with the most severe disease are not represented in the day 3 and 4 data.

### Histopathology and IHC analysis

**Figure 4:**
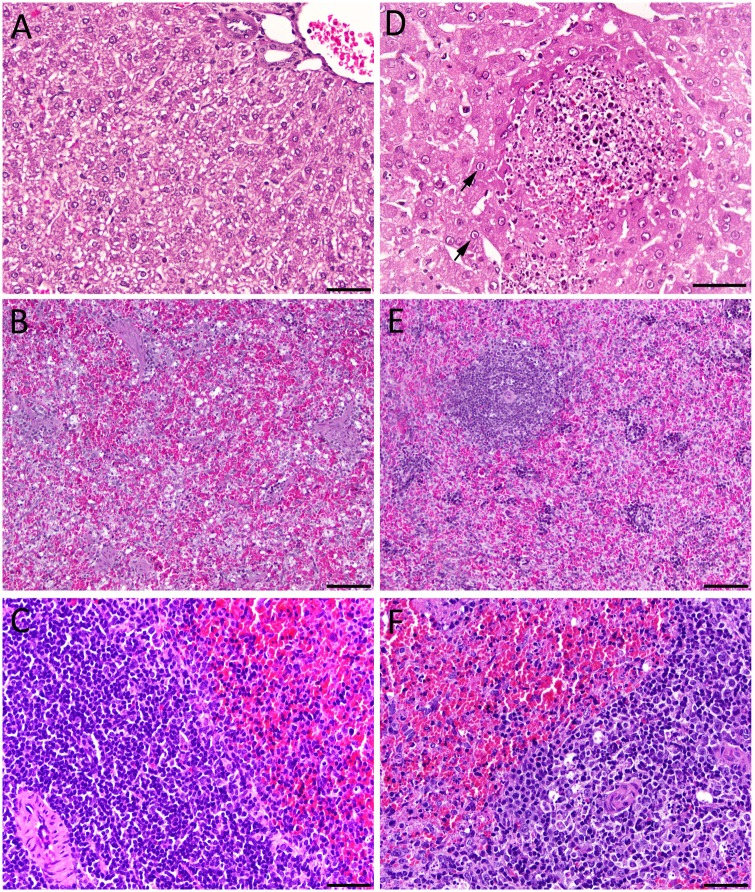
Histological findings in livers and spleens from RVFV-infected hamsters. A) Hamster liver section from sham-infected control animal showing normal liver histology, 400X, bar = 50 μm. B) Hamster spleen from sham-infected control showing normal red pulp, 200X, bar = 100 μm and C) white pulp, 400X, bar = 50 μm. D) 1 PFU, day 3 p.i. hamster liver (Fig. 3, half-filled triangle) showing acute hepatocellular necrosis and eosinophilic nuclear inclusions (arrows) in hepatocytes surrounding the area of necrosis 400X, bar = 30 μm. E) 10 PFU, day 2 p.i. hamster spleen (Fig. 2, open triangle) displaying diffuse erythroid depletion of the red pulp and lymphoid depletion of the white pulp, 200X, bar = 100 μm. F) 1 PFU, day 3 p.i. hamster spleen (Fig. 3, half-filled triangle) displaying diffuse lymphoid depletion of the white pulp. Heterochromatic cell fragments, indicative of apoptotic bodies and tingible body macrophages with cytoplasmic phagocytized apoptotic debris are scattered in the periarteriolar lymphoid sheath. 400X, bar = 50 μm. Hematoxylin and Eosin stain.

Histopathology and subsequent IHC analysis was performed on all collected tissues, as described above. Histologic examination of the livers from the 10 and 1 PFU challenged animals identified the liver as the primary target organ of infection. Overall, the main histologic lesion in the liver was randomly distributed multifocal acute hepatocellular necrosis with frequent eosinophilic intranuclear inclusions (Cowdry type A) in hepatocytes surrounding the areas of necrosis (Fig. 4B), which become apparent by day 2 and 3 p.i. for the 10 and 1 PFU challenged animals, respectively.

In general, the spleens of hamsters from both 1 and 10 PFU challenge groups exhibited a mild increase in lymphocyte area, and cellularity of the periarteriolar lymphoid sheath and lymphoid follicle. Erythrocyte depletion of the splenic red pulp, possibly due to splenic contraction, was detected in 2 of 4 animals in the 10 PFU group at day 1 p.i, with one animal also having white pulp (lymphoid) depletion (Fig. 4E). In the day 2 p.i. 10 PFU sacrifice group, 3 of 4 animals began to exhibit multifocal hepatocellular necrosis; one animal also had discernable erythroid and lymphoid depletion in the spleen (Fig. 4F). Comparatively, erythroid or lymphoid depletion was not observed in the 1 PFU challenge group until day 3 p.i. in 1 of the 3 surviving animals (Fig. 4F). Additionally, a significant amount of cell debris in the red pulp was observed, suggesting necrosis or apoptosis of lymphocytes and/or other circulating cells migrating through the splenic parenchyma. Of the hamsters sacrificed on day 4 p.i. only one animal had detectable white pulp depletion (data not shown). Little to no significant microscopic lesions was observed in pancreas, lung, brain, large intestine, kidney, adrenal gland, or eye tissues.

**Figure 5:**
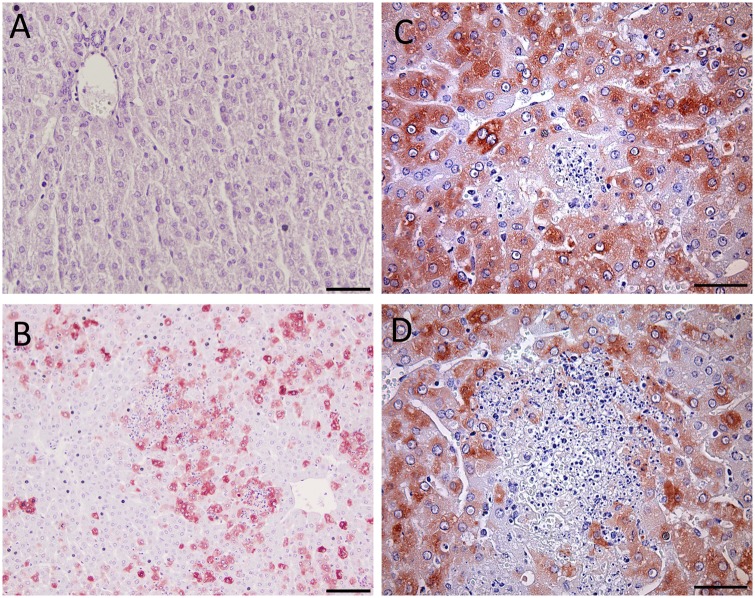
Immunohistochemistry of liver tissues from RVFV-infected hamsters demonstrates presence of viral antigen. A) Hamster liver section from sham-infected control animal, 400X, bar = 50μm. B) 10 PFU, day 2 p.i. hamster liver (Fig. 2, open circle) with 30–40% of hepatocytes exhibiting immunoreactivity for RVFV antigen, 20X, bar = 100 μm. C) 1 PFU, day 3 p.i. hamster liver (Fig. 3, half-filled triangle) with hepatocytes showing strong diffuse cytoplasmic immunoreactivity for RVFV antigen, 400X, bar = 50 μm. D) 1 PFU, day 4 p.i. hamster liver (Fig. 3, open upside-down triangle) with hepatocytes staining positive for RVFV antigen, 400X, bar = 50 μm. NovaRed stain with hematoxylin QS counterstain.

Successive IHC staining of the collected tissues generally demonstrated increased immunoreactivity in the liver with the most severe lesions. No immunoreactivity was observed in any of the animals challenged with 10 PFU at day 1 p.i., but by day 2 p.i. approximately 30–40% of hepatocytes examined showed multifocal to diffuse, and mild to strong cytoplasmic immunoreactivity for RVFV antigen (Fig. 5B); positive hepatocytes are in small groups or randomly distributed individual hepatocytes. The 1 PFU challenge group did not display any immunoreactivity until day 3 p.i., when IHC staining revealed most hepatocytes (approximately 90%) in the liver sections having strong, diffuse, cytoplasmic immunoreactivity for RVFV antigen (Fig. 5C). Occasional multifocal RVFV positive cells were present in the sinusoids and were interpreted as likely infected Kupffer cells (data not shown). On day 4 p.i. approximately 40–50% of hepatocytes exhibited a multifocal to diffuse, and mild to strong, cytoplasmic immunoreactivity for RVFV antigen (Fig. 5D); positive hepatocytes are in larger areas/groups of hepatocytes. The hepatocytes surrounding the areas of hepatocellular necrosis were positive for RVFV, but only rare cell debris was positive for viral antigen in the areas of necrosis (Fig. 5C). Inclusion bodies in the nuclei of hepatocytes, endothelial cell lining blood vessels and sinusoids, and biliary cells were not immunoreactive. As observed with the 10 PFU animals, no staining for RVFV antigen was observed in the spleen, brain, kidneys, lung, pancreas, adrenal gland, intestine, blood vessels, or eye in the hamsters challenged with 1 PFU.

## Discussion

Although previous studies have examined the susceptibility of hamsters to lethal RVFV infection [29–34], a more detailed description of the natural history is lacking. Here, we have characterized a model of s.c. RVFV infection in hamsters based on challenge with the ZH501 strain of the virus and discuss our findings in terms of other rodent RVFV models and severe cases of disease in humans. Consistent with earlier studies reporting a high degree of susceptibility, hamsters succumbed to a 10 PFU challenge with the ZH501 strain of RVFV within 2 to 3 days. By comparison, C57BL/6J mice challenged with 100× more PFU of the same virus stock succumbed in 3 to 6 days [36], underscoring the heightened sensitivity of hamsters to acute RVFV-induced disease.

As described by Smith and colleagues [17], we found that a wide variety of tissues supported RVFV infection in hamsters. Moreover, previous hamster studies utilizing RVFV describe viremia and elevated viral loads in liver, brain, and spleen tissues similar to our findings [29, 31, 34]. Based on our viral titer, serum ALT, histopathology, and IHC data, the liver was clearly the primary target for RVFV infection. The severe hepatocellular necrosis seen early during infection and the intense immunoreactivity of affected hepatocytes suggests that the hamsters were likely succumbing from fulminant hepatitis. This is in contrast the age-dependent gerbil RVFV infection model where liver involvement is minimal and encephalitis is believed to be the cause of death [37]. Marked elevation of serum ALT levels indicative of hepatocellular injury was observed in several hamsters in the 10 PFU challenge group that had substantial liver viral titers. In contrast, despite considerably high viral loads on day 4 in the livers of most of the hamsters challenged with 1 PFU, the ALT levels were not elevated. Because we did not perfuse the animals prior to tissue collection, contamination from virus present in the residual blood likely contributed to the elevation in the tissue viral loads. However, we suspect that delayed seeding of the liver may have resulted in slower replication of RVFV in the low-dose (1 PFU) challenge group, thereby affecting the kinetics of hepatocellular damage and subsequent release of ALT into the circulation. In mouse RVFV infection models, substantial liver viral titers have been observed as early as day 2 p.i., yet increases in serum ALT levels lag behind by approximately 1 day [17, 36, 38].

Although infectious RVFV was present in many tissues, histopathology was restricted primarily to the liver and, to a lesser extent, the spleen. The observed red and white pulp depletion of the spleen is similar to the pathology documented in the PTV hamster infection model wherein splenic necrosis involved both the red pulp and the lymphoid zone [15]. In contrast, PTV-infected C57BL/6 mice present with lesions that are more prominent in the white pulp [39]. RVFV infection in BALB/c mice displayed depletion of red pulp and lymphocyte apoptosis [17]. Although apoptotic bodies were visually identified, we did not perform a TUNEL assay or electron microscopy to confirm cellular apoptosis of the splenic white pulp.

The lack of RVFV antigen staining in tissues which contain high infectious viral loads and limited cellular damage, as observed in the spleen, could be due to a delay in the accumulation of detectable levels of antigen which may have reduced immunoreactivity, masking by prolonged exposure to the formalin preservative, or the sensitivity of the IHC staining. In the study by Smith et al., infectious RVFV was detected in the brain as early as day 3 p.i. yet antigen was not detected until day 6 p.i. and histological changes in the brain were not pronounced until day 8 p.i. [17]. A different study investigating chemotactic and inflammatory responses in mice reported that despite moderate amounts of necrotic debris observed in the spleen, viral antigen was not detectable in 20% of RVFV infected mice, and only very low level staining was observed in a small percentage of cells in the remaining 80% of the animals [38]. Additionally, in the related hamster PTV infection model, despite marked splenic necrosis, viral antigen was not detected [15].

Due to the inherent challenges of collecting samples from lethal cases of RVFV infection in remote regions of Africa and neighboring regions where the virus is endemic and medical infrastructure is often lacking, detailed description of RVF in humans is limited. The development of non-human primates (NHP) models of RVFV infection has facilitated investigations into the pathogenesis of the disease and the evaluation of potential antiviral therapies [14]. During severe infections in rhesus macaques, hemolytic anemia, extensive liver necrosis and possible disseminated intravascular coagulation (DIC) have been reported [40–42]. Despite limited histologic and IHC data, viremia, elevated serum ALT levels, and increased viral titers in the livers and spleens of fatally infected monkeys are consistent with our findings in RVFV-infected hamsters. Although both species develop significant lesions in the liver following RVFV challenge, the macaques exhibit a hepatocellular coagulative necrosis with cellular infiltrates not specifically observed in the hamster infection model [43]. Spleens from RVFV-infected rhesus macaque contained deposits of eosinophilic fibrin-like material in the red pulp of the spleen and a mild depletion of lymphocytes in the white pulp, similar to human infection, and our findings in hamsters infected with RVFV [40].

Although NHP models are considered the gold-standard when modeling RVF, they are cost-prohibitive and require special handling facilities. Thus, rodent models are better suited for initial stages of antiviral drug and vaccine development. Unlike NHP models, challenge of commonly used rodent species produces peracute disease and uniform lethality. The high mortality is favorable for antiviral and vaccine efficacy studies, but the often sublethal infection in NHPs is more representative of human infection wherein only a small percentage of those exposed progress to severe disease [3, 44–46]. Table 1 provides a comparison of the principal RVFV infection animal models in terms of general aspects one may want to consider to assist in selecting the most appropriate model for their research needs. These are only generalized guidelines as many factors such as the route of infection, dose and strain of challenge virus, and the age and strain of the animal species can affect the outcome of RVFV infection and associated disease.

**Table 1:** Comparison of RVFV animal models to the Syrian hamster model.

	**MODELS**					
**General Considerations**	Mouse	Rat ^a^	Hamster	Marmoset	AGM ^b^	Macaque
Lethality	High	Low-High	High	Moderate	Moderate	Low
Aerosol	Yes	Yes	ND	Yes	Yes	Yes
Cost	Low	Low	Low	High	High	High
Availability/Accessibility	High	High	High	Low	Low	Low
Mortality from Acute Disease	3–5 days	3–5 days	2–3 days	9–11 days	9–11 days	5–15 days
**Components of Human Disease**						
Biphasic Fever	No	No ^c^	ND	Yes	Single-phase	Yes
Hemorrhage	Yes	No	No	Yes	No	Yes
Petechia	No	No	No	No	No	Yes
Thrombocytopenia	Yes	ND	ND	NEI	NEI	Yes
Leukopenia	Yes	ND	ND	No	No	Yes
Lymphoid depletion	Yes	ND	Yes	ND	ND	ND
Lymphocyte apoptosis	Yes	ND	NEI	ND	ND	ND
Infection of macrophages	Yes	ND	NEI	ND	ND	ND
Coaugulopathy (DIC)	No	ND	ND	NEI	NEI	Yes
Hepatitis/Hepatocellular Necrosis	Yes	Yes	Yes	Yes	No	Yes
Retinitis	No	No	No	NEI	No	No
Delayed-Onset Encephalitis	Yes	Yes	Yes ^d^	Yes	Yes	No

^a^ Strain dependent

^b^ Aerosol challenge

^c^ Elevated body temperature prior to death

^d^ Ribavirin-treated animals

AGM, African green monkey; ND, not determined; NEI, not enough information

In summary, RVFV infection of hamsters most closely resembles the disease observed in mice, but with a more accelerated progression and without the development of encephalitis which can develop in mice. Although rapid lethality makes for an abbreviated therapeutic window and translation to the human condition difficult, the uniform lethality via low-dose inoculation with an acute, fulminant hepatic disease makes the hamster RVFV infection model a cost-effective system for evaluating experimental vaccines and antivirals to demonstrate initial proof-of-concept. More specifically, the hamster model is most useful for the evaluation of host-targeted interventions that are not active in the mouse, but do cross-react with the orthologous target in hamsters [28]. In addition, the ability to reliably produce a delayed neurologic disease when treating RVFV infection with ribavirin may prove useful for future studies investigating the role of ribavirin in late-onset neuroinvasion and associated encephalitis and the evaluation of potential neuroprotective countermeasures [13]. Infection by low volume intranasal or aerosol exposure should be evaluated to determine whether neurologic disease is favored under such exposure conditions, as has been demonstrated in mice and rats [18, 47]. This challenge route is highly relevant in terms of biodefense, as it would mimic respiratory route exposure that could occur through intentional release, and would likely produce a slower-progressing disease model.
